# Guidelines for Free-Energy Calculations Involving Charge Changes

**DOI:** 10.1021/acs.jctc.3c00757

**Published:** 2024-01-02

**Authors:** Drazen Petrov, Jan Walther Perthold, Chris Oostenbrink, Bert L. de Groot, Vytautas Gapsys

**Affiliations:** † Institute for Molecular Modeling and Simulation, Department of Material Sciences and Process Engineering, University of Natural Resources and Life Sciences, Vienna, Vienna 1190, Austria; ‡ Christian Doppler Laboratory for Molecular Informatics in the Biosciences, University of Natural Resources and Life Sciences, Vienna, Vienna 1190, Austria; § Computational Biomolecular Dynamics Group, Department of Theoretical and Computational Biophysics, Max Planck Institute for Multidisciplinary Sciences, Göttingen 37077, Germany; ∥ Computational Chemistry, Janssen Research & Development, Janssen Pharmaceutica N. V., Turnhoutseweg 30, Beerse B-2340, Belgium

## Abstract

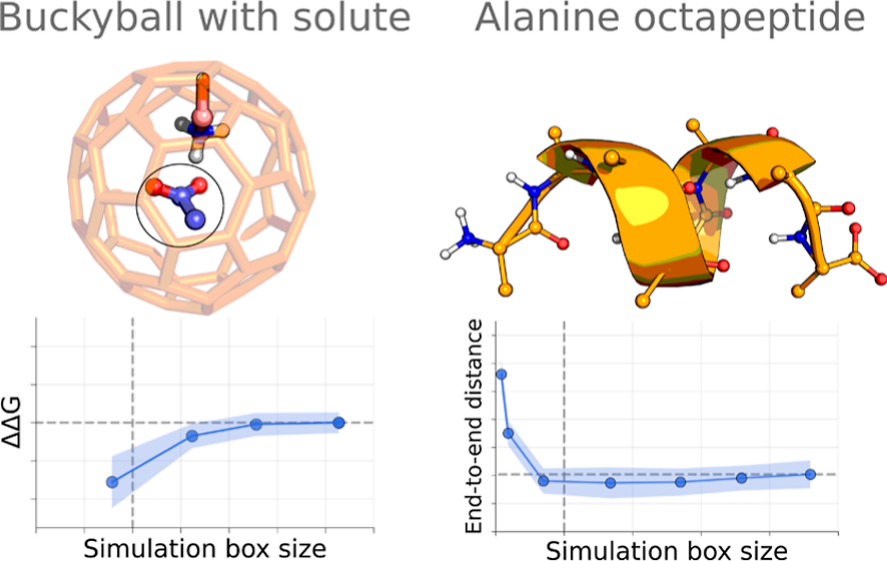

The Coulomb interactions in molecular simulations are inherently approximated due to the finite size of the molecular box sizes amenable to current-day compute power. Several methods exist for treating long-range electrostatic interactions, yet these approaches are subject to various finite-size-related artifacts. Lattice-sum methods are frequently used to approximate long-range interactions; however, these approaches also suffer from artifacts which become particularly pronounced for free-energy calculations that involve charge changes. The artifacts, however, also affect the sampling when plain simulations are performed, leading to a biased ensemble. Here, we investigate two previously described model systems to determine if artifacts continue to play a role when overall neutral boxes are considered, in the context of both free-energy calculations and sampling. We find that ensuring that no net-charge changes take place, while maintaining a neutral simulation box, may be sufficient provided that the simulation boxes are large enough. Addition of salt to the solution (when appropriate) can further alleviate the remaining artifacts in the sampling or the calculated free-energy differences. We provide practical guidelines to avoid finite-size artifacts.

## Introduction

For many years, the calculation of free-energy differences involving a net-charge change from molecular simulations has been a challenge due to finite-size effect artifacts. Because of the nanoscopic scale of most simulation systems, long-range electrostatic interactions are either truncated at a given cutoff and subsequently approximated by a reaction field beyond the cutoff^1,2^ or computed using lattice-sum methods.^3−6^ Both approaches are only approximations of the truly long-ranged Coulomb interactions, with different effects on the outcome of free-energy calculations. In particular, when net-charge changes are involved, the outcome will depend on either the size of the cutoff when using the reaction-field method or the size of the simulation box when using lattice-sum methods.

To obtain methodology-independent free-energy differences, two approaches have been established: (1) one may correct *a posteriori* for the artifacts that arise by computing corrections derived from the implicit solvent using Poisson–Boltzmann calculations^7−9^ or (2) one may avoid the occurrence of net-charge changes by transforming an appropriate ion simultaneously to the modification of interest, to construct an overall process that is charge neutral throughout.^10,11^ Note that both approaches still may require additional corrections for the type of summation over the discrete water molecules or for the Galvani potential of moving the particles over the water–vacuum interface.^7,12^

Recently, the size of potential artifacts for various kinds of binding free energies was systematically studied for a set of model host–guest systems. Using both a cutoff scheme and a lattice-sum approximation to the long-range electrostatic interactions, and applying this to both an alchemical approach and a path sampling approach, the size of remaining corrections in the absence and presence of additional salt in the solution was quantified.^13^ It was found that significant corrections remained for the various approaches. However, the coalchemical approaches, in which an ion in solution is perturbed simultaneously to the ligand perturbation in an alchemical approach, were partly set up such that no net-charge change occurred, but a constant overall non-neutral charge was kept in the system. In the applied lattice-sum method, this is compensated by a neutralizing background charge which was previously shown to be inappropriate in such free-energy calculations.^11,14^ In addition, results from the current work support this observation showing that the artifacts are larger for the cases with the background charge present.

Free-energy calculations were performed on a buckyball model system with varying charge states and positively or negatively charged ligands.^15,16^ Here, we expand the work of Öhlknecht et al. to include different simulation box sizes and alternative setups of counterions and additional salt. Furthermore, we investigate how the use of approximate electrostatics influences the sampling of an overall neutral, zwitterionic peptide. Box-size-dependent sampling was previously described for this model system but with relatively short simulation times and without the use of additional salt.^13^

In the current work, we set out to investigate whether free-energy differences and molecular ensembles sampled by molecular dynamics simulations when treating long-range electrostatics with the lattice summation methods require corrections, as suggested earlier.^7,8,12−14,16,18−22^ In particular, would corrections be required for the ensembles (and the corresponding free-energy differences) when the system carries no overall charge at all times? Answering these questions allowed us to formulate a rule-of-thumb for the box-size generation, which ensures a sufficient solvation layer to avoid unwanted electrostatic artifacts.

## Methods

### Buckyball Simulations and Analysis

#### Simulation Setup and Parameters

**Figure 1:**
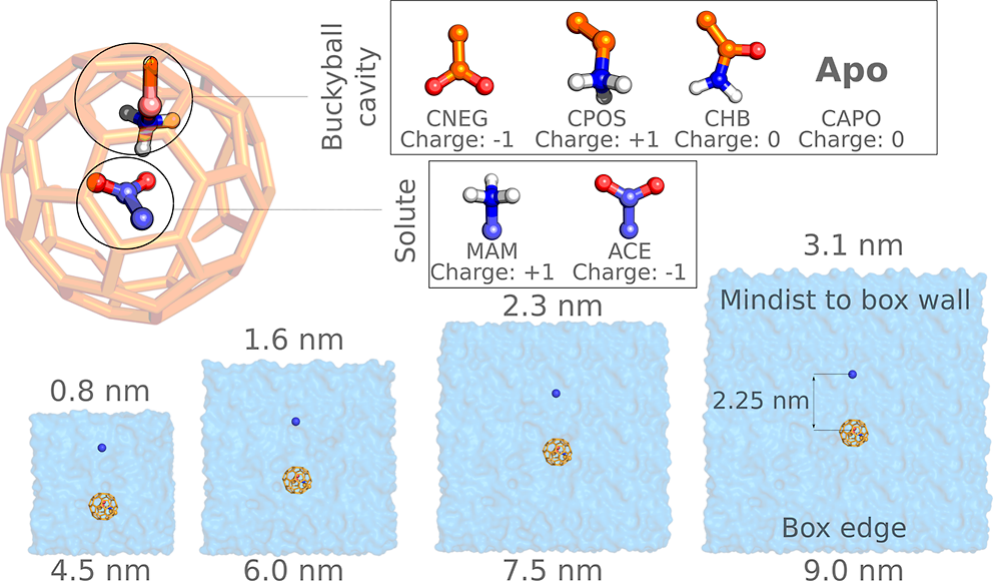
Summary of the alchemical free-energy calculation set up for the charge modifications in the buckyball systems. Four buckyball types (CNEG, CPOS, CHB, and CAPO) and two types of solutes (MAM and ACE) were probed. The simulations were performed in cubic boxes of four different sizes. During an alchemical transition, the electrostatic component of the solute was coupled to the system, while electrostatic interactions of an ion were simultaneously decoupled/perturbed.

All the simulations reported in this work were performed with the GROMACS^23^ 2018 version. The structures and topologies for buckyball systems were taken from Öhlknecht et al.^13^ and are summarized in Figure 1. Ligand parameters in GROMOS 53A6 force field were taken from ref (24) with an improper dihedral in the formate group of the CNEG molecule set to type 1 with a reference value of 0°, as described by Reif & Oostenbrink.^16^ The two solutes acetate (ACE) and methylammonium (MAM) were placed in the buckyballs containing chemical groups defined as follows: negative with the net charge of −1*e* (CNEG), positive with the net charge of +1*e* (CPOS), hydrogen-bonding neutral (CHB), and apo buckyball (CAPO) (Figure 1). In addition, ACE and MAM in solution without a buckyball were simulated as well.

The systems were placed in cubic boxes with an edge length of 4.5, 6.0, 7.5, and 9.0 nm. For ACE and MAM solvation in water without buckyball, we used boxes with edge lengths of 3.25, 4.5, 5.5, and 6.5 nm. SPC^25^ water was used to solvate the created boxes. To enable alchemical coupling/decoupling of the solute by retaining a neutral charge of the simulation box, an ion was added to the system. This additional ion was decoupled/coupled from the system together with the ACE or MAM following the direction described in Table 1. The distance between the ion and nitrogen in MAM or carboxyl carbon in ACE was restrained at 2.25 nm with a force constant of 1000 kJ mol^–1^ nm^–2^. Furthermore, to probe the effects of salt, another set of systems was prepared analogous to those described above with addition of 0.5 M sodium and chloride ions.

**Table 1:** Summary of the System Setup for the Free-Energy Calculations^a^

	buckyball	solute	*q*_bucky_	*q*_solute_^A^ → *q*_solute_^B^	*q*_ion_^A^ → *q*_ion_^B^	*q*_overall_^A^ → *q*_overall_^B^
(13)		ACE		0 →–1	–1 → 0	–1 →–1
(13)	CAPO	ACE	0	0 →–1	–1 → 0	–1 →–1
(13)	CHB	ACE	0	0 →–1	–1 → 0	–1 →–1
(13)	CNEG	ACE	–1	0 →–1	–1 → 0	–2 →–2
(13)	CPOS	ACE	+1	0 →–1	–1 → 0	0 → 0
(13)		MAM		0 → +1	+1 → 0	+1 → +1
(13)	CAPO	MAM	0	0 → +1	+1 → 0	+1 → +1
(13)	CHB	MAM	0	0 → +1	+1 → 0	+1 → +1
(13)	CNEG	MAM	–1	0 → +1	+1 → 0	0 → 0
(13)	CPOS	MAM	+1	0 → +1	+1 → 0	+2 → +2
this work		ACE		0 →–1	0 → +1	0 → 0
this work	CAPO	ACE	0	0 →–1	0 → +1	0 → 0
this work	CHB	ACE	0	0 →–1	0 → +1	0 → 0
this work*	CNEG	ACE	–1	0 →–1	+1 → +2	0 → 0
this work	CPOS	ACE	+1	0 →–1	–1 → 0	0 → 0
this work		MAM		0 → +1	0 →–1	0 → 0
this work	CAPO	MAM	0	0 → +1	0 →–1	0 → 0
this work	CHB	MAM	0	0 → +1	0 →–1	0 → 0
this work	CNEG	MAM	–1	0 → +1	+1 → 0	0 → 0
this work*	CPOS	MAM	+1	0 → +1	–1 →–2	0 → 0
this work, salt (+1)	CNEG	ACE	–1	0 → 1–	0 → +1	0 → 0
this work, salt (−1)	CPOS	MAM	+1	0 → +1	0 →–1	0 → 0

a Free-energy calculations in this work were carried out following a consistent protocol (details in the Methods section) using the topologies summarized in the table. The upper part of the table corresponds to the scheme used in ref (13) where the overall charge is conserved during the alchemical transformation, yet the simulation box is not necessarily neutralized. The lower part of the table summarizes an approach to setting up the perturbations such that the overall neutral system is retained at all times during the transformation. The entries with an asterisk mark topologies that were used in the simulations without salt only. For these cases, simulations with salt had different topologies ensuring stable molecular dynamics runs: topologies are listed in the last two lines of the table.

During free-energy calculations, only the charges of the solute and the coalchemical ion were switched on/off. First, the systems were energy-minimized with ACE/MAM in their electrostatically decoupled state. Afterward, for each simulation case, 5 independent 10 ns equilibrations of this state were performed. For the cases with 0.5 M salt, the ions were added to thus equilibrated snapshots, and an additional 2 ns equilibration was performed. Finally, the equilibrated configurations were used to start 5 independent free-energy calculations for every simulation scenario. An equilibrium free-energy perturbation protocol was used by stratifying the alchemical path into 11 discrete equidistantly spaced λ-states. A 10 ns simulation at each λ window was performed. The final analysis was performed using the alchemical analysis tool^26^ by discarding the first 2 ns as equilibration time. The multistate Bennett acceptance ratio^27^ estimator was used to obtain the final free-energy differences. We ensured that free-energy estimates in this case did not depend on the particular choice of the estimator (Table S1).

The equilibration simulations were performed under *NPT* conditions, where a velocity rescaling thermostat^28^ with 0.1 ps time constant kept the temperature at 300 K and a Parrinello–Rahman barostat^29^ with a time constant of 5 ps was used to keep the pressure at 1 bar. The canonical ensemble was sampled for the free-energy calculations. Long-range electrostatics were treated using particle mesh Ewald (PME)^3,4^ with a real space cutoff of 1 nm, PME order of 6, Fourier grid spacing of 0.1 nm, and relative strength at the cutoff of 10^–5^. The van der Waals interactions were cut off at 1 nm. For the equilibrium simulations, a dispersion correction was applied to energy and pressure, while for the free-energy calculations, an energy correction was applied.

#### Analysis

To calculate the minimal distance to the box wall, we determined the smallest distance between any atom of the solute and any atom of its periodic images. The minimum value of this smallest distance observed during the simulation was subsequently halved to obtain the minimal distance to the box wall. The whole construct of buckyball, ACE, or MAM and the restrained ion were considered as a solute for the distance calculation.

The free-energy differences in buckyballs were calculated as means over 5 simulations. Uncertainties are reported as a 95% confidence interval computed from the standard error of 5 simulation repeats assuming a normal distribution of the free-energy values. To visualize the box-size dependency, we depict the free-energy differences between the value obtained for a corresponding box and the value in the largest simulated box.

### Peptide Simulations and Analysis

#### Simulation Setup and Parameters

**Figure 2:**
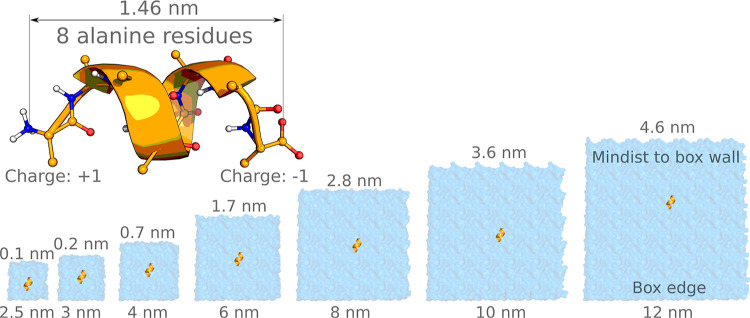
Summary of the alanine octapeptide simulations. The peptide was simulated in its zwitterionic form in cubic boxes of 7 different sizes.

Alanine octapeptide with charged termini residues was parametrized with the GROMOS 54A7^30^ force field. The initial structure was generated using pmx^31^ to form a helical secondary structure. The peptide was placed in cubic boxes of different sizes with an edge length of 2.5, 3, 4, 6, 8, 10, and 12 nm (Figure 2). Subsequently, the structure was solvated with SPC water. In addition to the setup described above, we have also prepared a system following the same steps and adding 0.15 M salt of chloride and sodium ions.

After the energy minimization, the systems were simulated by running 50 independent replicas of 1 μs each for both setups (with salt and without) and for each box size. Most of the simulation parameters were retained identical to the buckyball simulations, except that the PME order was set to 4 and the Fourier grid spacing was set to 0.12 nm. Also, the pressure of 1 bar was kept with the Parrinello–Rahman barostat^29^ with a time constant of 5 ps. The dispersion correction was applied to both the energy and pressure.

#### Analysis

The first 100 ns was discarded from each generated trajectory as equilibration time. As for the buckyball systems, we calculated the minimal distance to the box wall by computing the smallest distance between any peptide atom and any atom of its periodic images. Subsequently, we halved the minimum value of this smallest distance observed during the simulation to obtain the minimal distance to the box wall. The end-to-end distance for the peptide was computed between the nitrogen atom of the first residue and the carboxyl carbon atom of the last residue. The free-energy profiles were computed from population counts projected on the end-to-end distance as a reaction coordinate. Variance-minimizing superpositioning^32^ was used for the representative subensembles. The uncertainties for the free-energy profiles are reported as 95% confidence intervals calculated from the standard errors of 50 independent replicas for each simulated case and assuming the normality of the Δ*G* distribution. The significance of the free-energy differences and mean end-to-end distance between extended and collapsed states was determined by calculating the difference between the corresponding box and the largest box and evaluating whether zero falls outside of the 95% CI.

For the peptide orientation analysis, the vector from the first residue nitrogen atom to the last residue carboxyl carbon atom was calculated. The orientation of the vector was projected onto the spherical coordinates.

### Free-Energy Charge Corrections

Charge corrections were calculated according to^9,13,16^ and potentially consist of three separate terms: (1) a correction for inaccurate solvent polarization Δ*G*_pol_, (2) a correction for non-Coulombic direct nonsolvent interactions Δ*G*_dir_, and (3) a correction for the potential from discrete solvent molecules Δ*G*_dsm_.

The current work focuses on Δ*G*_pol_ and Δ*G*_dir_, which are calculated by comparing the solute–solvent and solute–solute interactions as they are computed during the simulation, under periodic boundary conditions, to the ideal case involving purely Coulombic interactions at nonperiodic conditions.

Δ*G*_pol_ was calculated using the dGslv_pbsolv program^13^ included in the GROMOS++ simulation package. This program employs a finite difference (FD) Poisson equation solver^33,34^ capable of handling periodic boundary conditions in combination with a fast Fourier transform (FFT) Poisson equation solver capable of handling RF and LS schemes.^16,35,36^ It compares the solvation free energy under periodic boundary conditions to its nonperiodic counterpart. The contribution of correcting for an incorrect dielectric constant of the SPC water model (compared to the experimental value of the solution) to Δ*G*_pol_ was not taken into account. Note that when such a contribution is included to Δ*G*_pol_ (using 78 as an arguably too large of a value for the dielectric constant of the salt solution), this leads to a constant (box-size-independent) offset of the obtained charge corrections of up to 6 kJ/mol for the systems ACE in CNEG and MAM in CPOS.

Δ*G*_dir_ was calculated by evaluating the electrostatic potential energy of the solute under periodic (same as the simulation setup) and nonperiodic (infinite cutoff) conditions at both end states of the perturbation. Both Δ*G*_pol_ and Δ*G*_dir_ were calculated as averages over 20 snapshots taken equidistantly in time from the end-state simulations (λ = 0 and λ = 1).

Δ*G*_dsm_, also referred to as Δ*G*_C_ in earlier work,^8^ involves a contribution for summation over discrete water molecules and a contribution for the transfer of an ion over the vacuum–liquid interface.^7,8^ As the net-charge change in the system is zero, the former contribution is equal to 0, while the latter term is negligible for ions that are small in relation to the box volume. Δ*G*_dsm_ was therefore not taken into account in this work.

## Results and Discussion

### Coalchemical Free-Energy Calculations

In the first part of this study, we focus on free-energy calculations based on an alchemical approach. To probe whether the finite-size effects in such simulations are indeed significant and can be detected, we have constructed a set of buckyball systems analogous to those described earlier^13,15,16^ (detailed description in the Methods section and Figure 1). The simulation setup allows one to evaluate the transfer free energy of a solute, MAM (positively charged) or ACE (negatively charged), from vacuum into a buckyball.

During an alchemical transition of the MAM or ACE moiety, a coalchemical ion was perturbed such that a constant overall charge of the simulation box was retained. There are multiple ways to construct a scheme for an alchemical transition that keeps the charge constant. The setup of Öhlknecht et al.^13^ was designed in such a way that 8 out of 10 considered systems were not neutralized, namely, they carried an overall charge of −1*e*, + 1*e*, or +2*e*, as summarized in Table 1. We repeated these simulations in this work. Additionally, we used a setup where the coalchemical ion was assigned a charge (at both end states, which can also include 0 charge, Table 1) such that the simulation box remained neutral at all times during an alchemical transition (akin to the double-system/single-box setup^10^). Additionally, we performed the same set of simulations in which an additional 0.5 M sodium chloride was added following the setup by Öhlknecht et al.^13^ These simulations are denoted as “salt”.

The finite-size effects on the thermodynamics of the system can be read out by inspecting free-energy differences in simulation boxes of varying sizes.^9,37,38^ In the largest box, the periodicity-induced artifacts should be the least pronounced. Thus, by observing the trend in the Δ*G* change when going from smaller to larger boxes, we estimated the magnitude of such artifacts. Having performed simulations in the boxes of different sizes, we calculated the free-energy difference of switching on the electrostatic interactions of a solute with the environment while simultaneously perturbing the electrostatic interactions of the coalchemical ion.

#### Buckyballs without Salt

**Figure 3:**
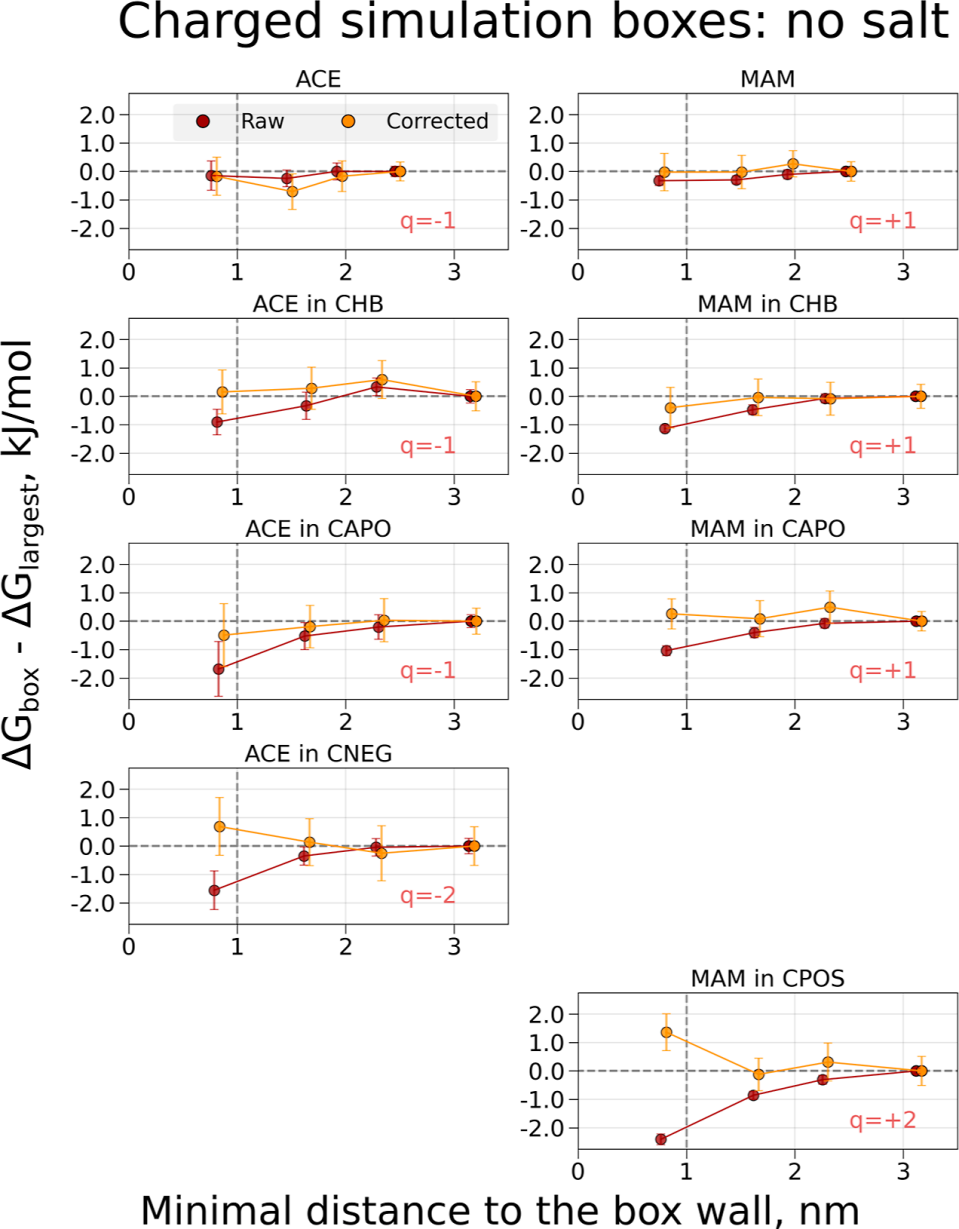
Free-energy differences and corrections for coupling electrostatic interactions of the ACE and MAM ligands and decoupling/coperturbing an ion in solution. The charge modifications were performed to retain a constant overall charge in the simulation box. The topology setup retaining a constantly charged box was used. The systems were solvated in water, and no additional salt was added. Simulations were performed in cubic boxes of varying size: the values on the *x*-axis denote the minimal distances for the buckyball-solute-ion to the cell wall for every box size; the values on the *y*-axis denote the Δ*G* differences between the values obtained in the individual box and the largest considered box. The darker-colored symbols indicate raw calculated free energies, while the light-colored symbols mark the free-energy values with the corrections added. The symbols for the corrected values are offset by 0.05 nm along the *x*-axis for visualization purposes.

**Figure 4:**
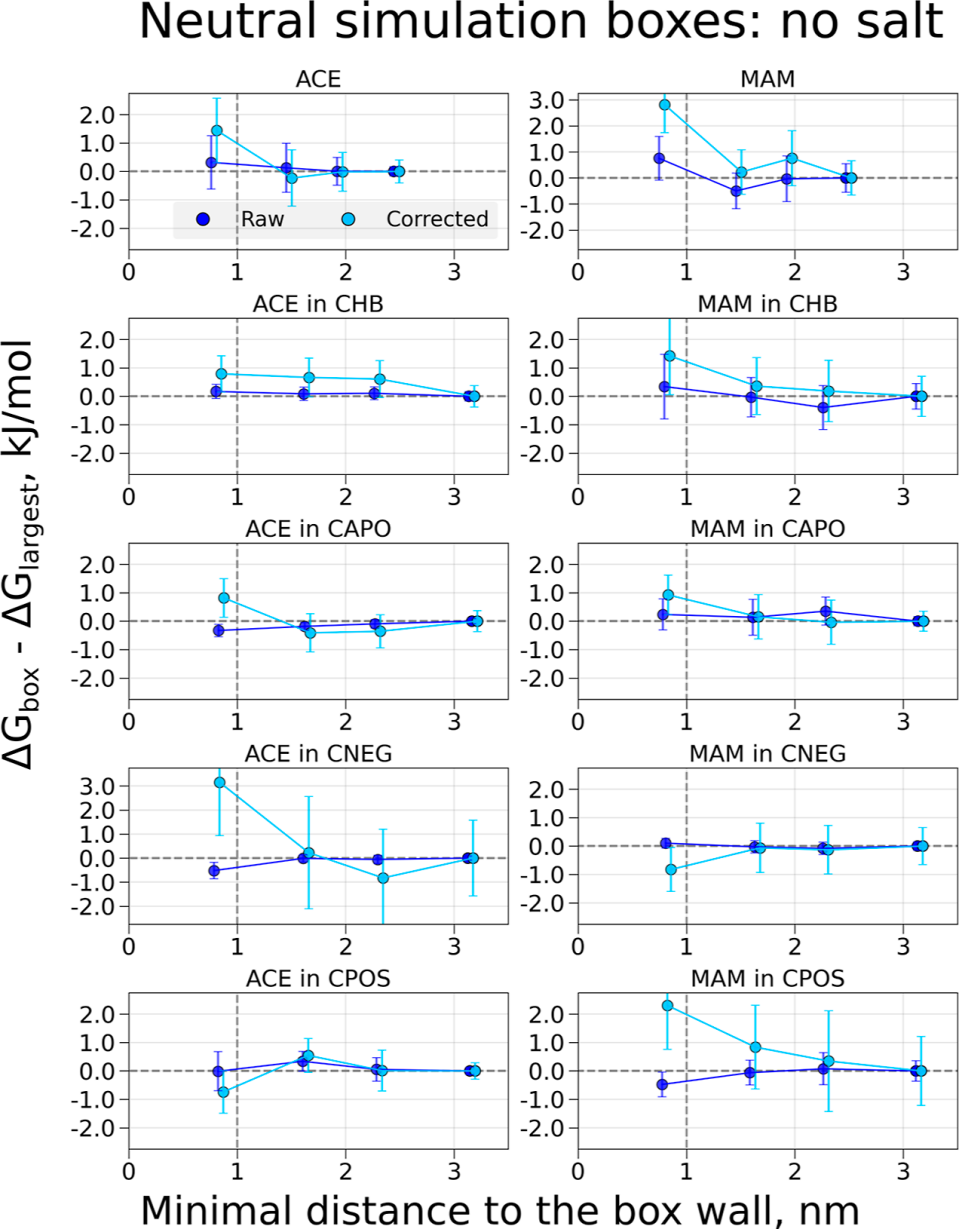
Free-energy differences and corrections for coupling electrostatic interactions of the ACE and MAM ligands and decoupling/coperturbing an ion in solution. The charge modifications were performed to retain a constant overall charge of the simulation box. The topology setup retaining a neutral simulation box was used. The systems were solvated in water and no additional salt was added. Simulations were performed in cubic boxes of varying size: the values on the *x*-axis denote the minimal distances for the buckyball-solute-ion to the cell wall for every box size; the values on the *y*-axis denote the Δ*G* differences between the values obtained in the individual box and the largest considered box. The darker-colored symbols indicate raw calculated free energies, while the light-colored symbols mark the free-energy values with the corrections added. The symbols for the corrected values are offset by 0.05 nm along the *x*-axis for visualization purposes.

First, we investigate the changes in the calculated Δ*G* for the simulations in pure water without additional salt. When exploring the box-size-dependent trends, it is convenient to monitor the difference between the Δ*G* value in a box of a given size and the Δ*G* calculated in the largest explored box (Figures 3 and 4). This way, any deviation from zero indicates a finite-size artifact due to a simulation box that is too small.

The raw free energies for the charged simulation boxes show considerable deviations from zero for smaller box sizes (Figure 3, the Δ*G* values are collected in the Supporting Information Tables S2–S9). Generating boxes that are large enough to ensure at least a 1 nm distance to the box wall may not be a sufficient rule-of-thumb to avoid finite-size effects for the non-neutral systems. The largest finite-size artifact is observed for the MAM in the CPOS case (*q* = +2*e*).

Corrections of the free energies were also calculated, and the free-energy differences of the corrected values are shown in light-colored symbols in Figure 3, with the corrections themselves shown in Figure S1 (also in the Supporting Information Tables S10–S13). In most cases, the corrections keep the difference Δ*G*_box_ – Δ*G*_largest_ close (ACE/MAM) or bring it closer to zero (ACE/MAM in CHB, ACE/MAM in CAPO, and ACE in CNEG), such that the corrected free-energy values are within the respective error estimates from zero. This suggests that the corrections indeed remove the finite-size artifact. While varying less than the raw free-energy values, the corrected Δ*G* values of MAM in CPOS still show significant differences among the calculations in boxes of different sizes.

If the simulation boxes are kept neutral during the alchemical transformation, the raw free-energy values in Figure 4 vary much less with the box size than for the simulation boxes with a nonzero overall charge. We observe deviations from zero only for the smallest explored box size of ACE in CAPO, ACE in CNEG, and MAM in CPOS, with values well below 1 kJ/mol.

After applying the corrections for the neutral simulation boxes, the variation in Δ*G*_box_ – Δ*G*_largest_ remains within 1 kJ/mol for most simulations, with notable exceptions being the case for systems ACE in CNEG and MAM in CPOS, for which the corrections in the smallest simulation boxes rather seem to increase the deviation from zero. Seemingly, correcting free-energy differences that are already devoid of artifacts leads to the addition of a considerable amount of statistical noise for the cases where buckyball and ligand complexes acquire a charge of −2*e* and +2*e*. The Δ*G* correction values can be found in Figure S1. As expected, all corrections tend toward zero as the box size increases. The corrections for charged and neutral simulation boxes tend to follow very similar trends.

All in all, while the corrections might help to remove finite-size electrostatic artifacts for charged simulation boxes, they may also not be sufficient or may even have an adverse effect of introducing artifacts on their own for neutral simulation boxes. For such cases, i.e., overall neutral simulation boxes where the electrostatic artifacts can be expected to be negligible, the noise in the calculation of the corrections becomes more relevant than the remaining artifacts. This is potentially due to the choice of the calculation settings, e.g., the grid size in the PB calculations or in the lattice-sum method (leading to numerical inconsistencies), and the general assumption that an implicit solvent calculation captures the most relevant corrections for the solute–solvent interactions.

#### Buckyballs with Salt

**Figure 5:**
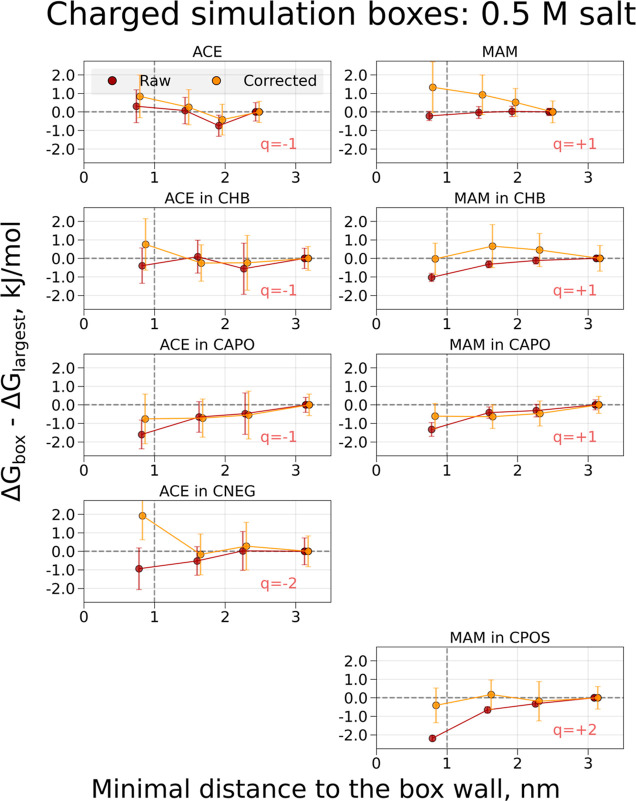
Free-energy differences and corrections for coupling electrostatic interactions of the ACE and MAM ligands and decoupling/coperturbing an ion in solution in systems with 0.5 M salt. The charge modifications were performed to retain a constant overall charge of the simulation box. The topology setup retaining a constantly charged box was used. The system was solvated in water and 0.5 M salt (sodium and chloride) was added. Simulations were performed in cubic boxes of varying sizes: the *x*-axis denotes the minimal distances for the buckyball-solute-ion to the cell wall for every box size; the values on the *y*-axis denote Δ*G* differences between the values obtained in the individual box and the largest considered box. The darker-colored symbols indicate raw calculated free energies, while the light-colored symbols mark the free-energy values with the corrections added. The symbols for the corrected values are offset by 0.05 nm along the *x*-axis for visualization purposes.

**Figure 6:**
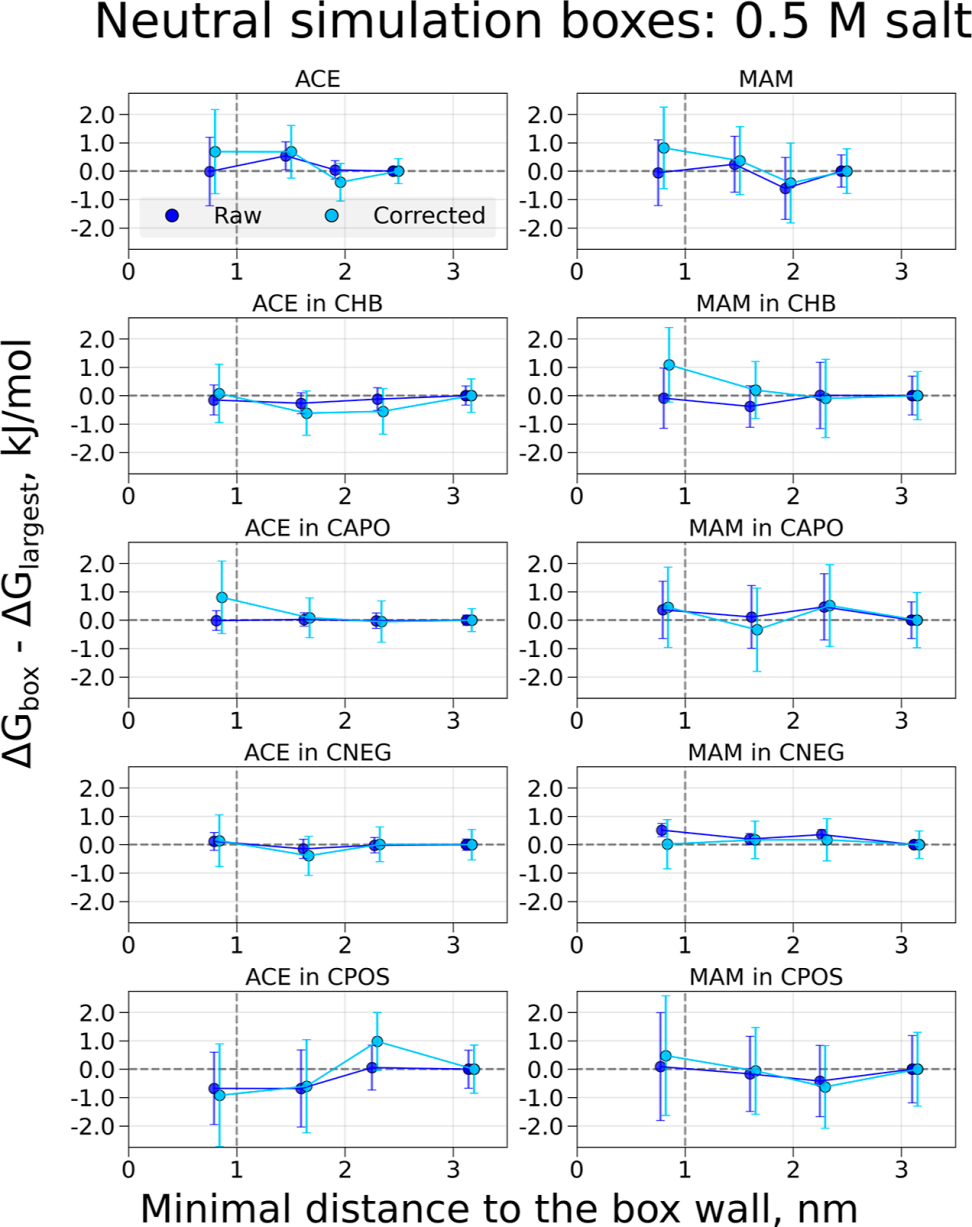
Free-energy differences and corrections for coupling electrostatic interactions of the ACE and MAM ligands and decoupling/coperturbing an ion in solution in systems with 0.5 M salt. The charge modifications were performed to retain a constant overall charge of the simulation box. The topology setup retaining a neutral simulation box was used. The system was solvated in water and 0.5 M salt (sodium and chloride) was added. Simulations were performed in cubic boxes of varying sizes: the *x*-axis denotes the minimal distances for the buckyball-solute-ion to the cell wall for every box size; the values on the *y*-axis denote Δ*G* differences between the values obtained in the individual box and the largest considered box. The darker-colored symbols indicate raw calculated free energies, while the light-colored symbols mark the free-energy values with the corrections added. The symbols for the corrected values are offset by 0.05 nm along the *x*-axis for visualization purposes.

Addition of 0.5 M NaCl salt did not significantly impact the trends of calculated Δ*G* values across the boxes of varying sizes (Figures 5 and 6). For charged simulation boxes, the raw data continue to show a box-size dependency, which can be removed by adding the appropriate corrections in all cases, except for the smallest box size of system ACE in CNEG. For the neutral simulation boxes, the raw Δ*G* values are already quite stable over the different box sizes, with deviations within 1 kJ/mol throughout. Adding corrections (Figure S2) to these calculations mostly adds statistical noise to the corrected values. Similar to the systems without salt, the corrections would deviate from zero if correcting for dielectric constant is included, however, to a smaller extent with the correction values not exceeding 3 kJ/mol even in most extreme examples of ACE in CNEG and MAM in CPOS systems. Note, however, that the definition of a coalchemical ion is different for these two systems with and without ions, which may affect the size of this correction term.

### Polyalanine Octapeptide Simulations

As demonstrated in the previous section, free-energy differences calculated with alchemical simulations may require corrections for particularly small boxes for systems carrying an overall charge. We have further set out to explore whether similar electrostatic finite-size artifacts could significantly manifest in plain (nonalchemical) molecular dynamics simulations. To address this, we chose to investigate an alanine octapeptide with charged termini (Figure 2): a molecular system for which finite-size effects have been reported to strongly affect conformational equilibria.^17^

#### Peptide Conformations

Similar to the case of the alchemical buckyball study, here we have performed molecular dynamics simulations of the peptide in cubic boxes of varying sizes, as summarized in Figure 2.

Due to its flexibility, the peptide’s minimal distance to the box wall varies substantially over the course of the simulation (Table 2). The smallest distance to the box wall in the boxes with edge lengths of 2.5, 3, and 4 nm is below 1 nm, i.e., the rule-of-thumb distance that we had marked in the previous buckyball analysis.

**Table 2:** Minimal Distances to the Box Wall^a^

box edge length	init	avg	min
2.5	0.6	0.5	0.1
3	0.8	0.8	0.2
4	1.3	1.3	0.7
6	2.3	2.3	1.7
8	3.3	3.3	2.8
10	4.4	4.2	3.6
12	5.4	5.2	4.6

a The distances were calculated as half of the minimal distance to the periodic image of the peptide. The “init” column corresponds to the minimal distance to the box wall for the starting helical peptide conformation. “avg”: minimal distance to the box wall averaged over all the simulations performed in a corresponding box. “min”: minimal distance to the box wall calculated by considering the smallest observed distance over all the simulations performed in a corresponding box. All values are in nm.

**Figure 7:**
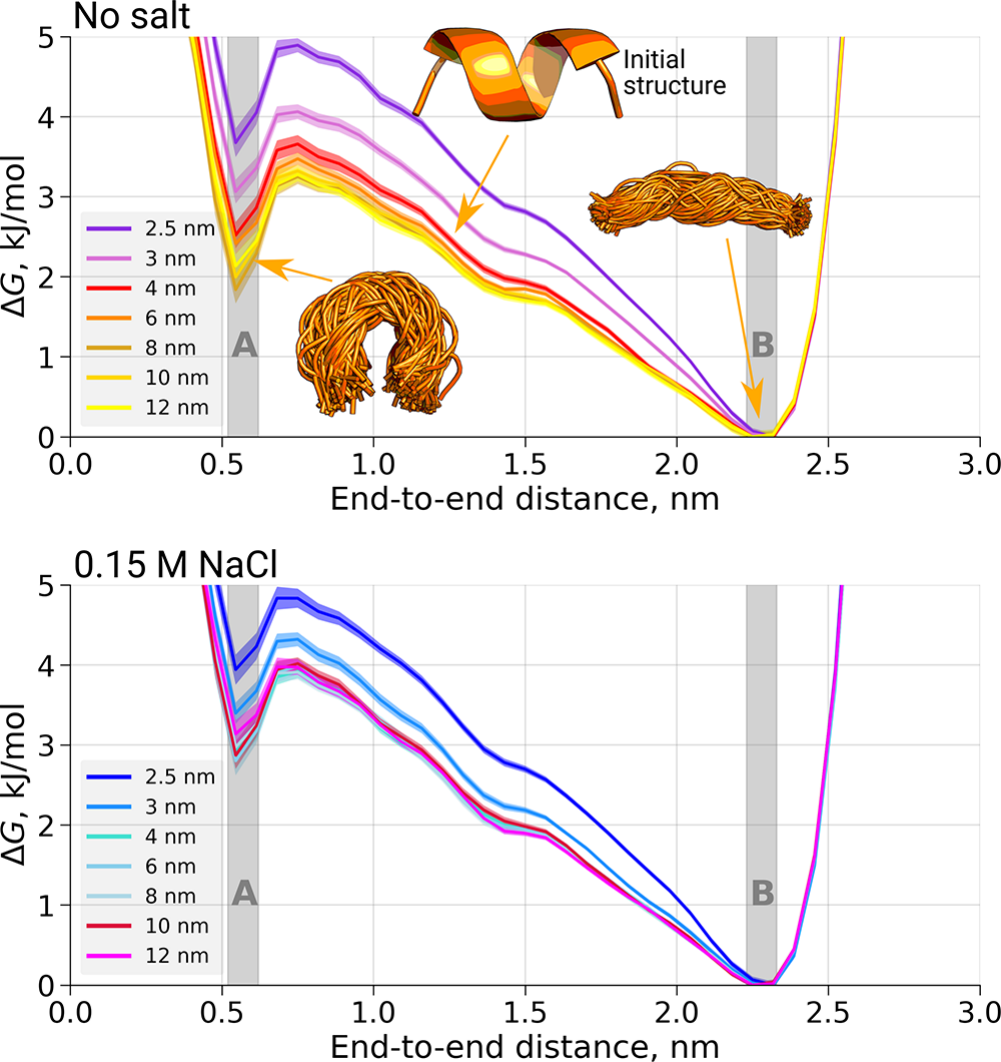
Free-energy profiles for the alanine octapeptide simulations. The top figure corresponds to the simulations without salt, and the bottom figure depicts simulations with 0.15 M NaCl. The regions of the two minima marked as **A** and **B** are considered for further analysis in Figure 8. Shaded areas correspond to the 95% confidence intervals estimated from independent simulations.

Reaching 50 μs of sampling time in each of the boxes allowed us to sufficiently converge the free-energy profiles along the peptide’s end-to-end distance coordinate (Figure 7). The overall trend in the profiles is independent of the box size: the initially helical structure is quickly lost, and extended conformations are predominantly sampled (marked as **B** in Figure 7). Another free-energy minimum, stabilized by the salt bridge between the charged termini, emerges at the shorter region of the end-to-end distances (marked as **A** in Figure 7).

**Figure 8:**
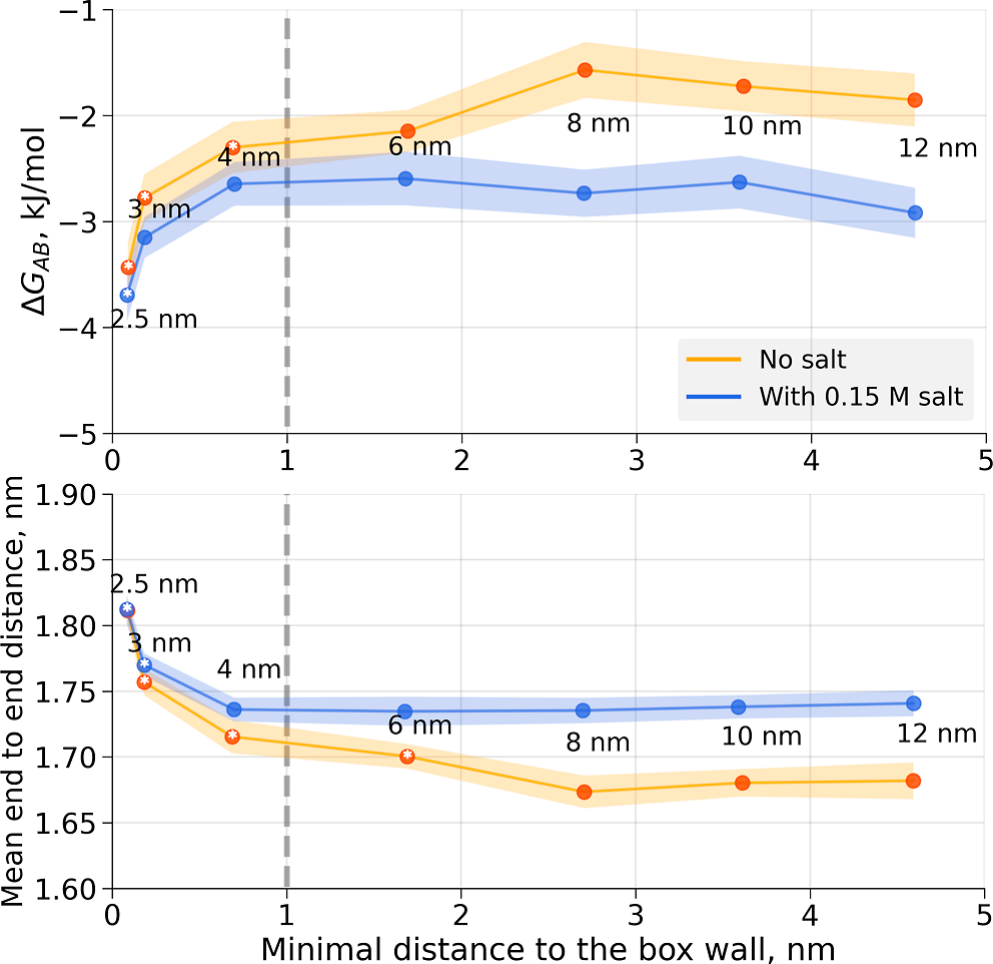
Δ*G*_AB_ and mean end-to-end distance dependence on the simulation box size. The top panel depicts the free-energy difference between the two conformational minima marked in Figure 7. The bottom panel shows the mean end-to-end distance. Those cases for which Δ*G*_AB_ or mean end-to-end distance differs significantly from the respective observable in the largest box are marked by *. Shaded areas correspond to the 95% confidence intervals estimated from independent simulations.

With respect to the varying box sizes, the free-energy profiles show differences for the smaller boxes (an edge length of 2.5, 3.0, and 4.0 nm for the simulations without salt). For the simulation in a box with an edge of 6 nm, the free-energy profile seems to be more similar to the larger box sizes, and the profile at the free-energy minima already does not significantly differ from the largest simulation box. For the simulations in 0.15 M NaCl, only the smallest box size (edge length of 2.5 nm) leads to a significantly different free-energy profile at the free-energy minimum compared to the largest box. The relative populations in the two minima (**A** and **B**) can be monitored to quantify the box-size dependence of the conformational preferences (Figure 8 top). To obtain the relative populations in **A** and **B**, we counted the peptide conformers in the respective regions in the simulated trajectories. It appears that the smallest boxes for which Δ*G*_AB_ significantly differs from the value calculated in the largest simulation box (marked by a *) do not ensure even a 1 nm minimal distance between the solute and the box wall. Note that the *x*-axis on this figure shows the minimal distance to the box wall as half of the smallest observed distance between the peptide’s periodic images.

Another observable that captures the contribution of all of the conformations of the sampled ensemble (in contrast to the **A** and **B** basins only) is the mean end-to-end distance (Figure 8 bottom). Similar to the observation from the Δ*G*_AB_ trend, the smallest boxes do not provide a sufficient distance between the periodic images to remove finite-size electrostatic artifacts. In this case, for the simulations without salt, an even larger box size allowing for more than 1.5 nm of the minimal distance to the box wall would be required.

The addition of a physiological concentration of salt (0.15 M NaCl in the current simulations) reduces the finite-size artifacts due to long-range electrostatics, as mobile charges contribute to the solute charge screening and reduce permanent and transient dipoles in the simulation box.^39^ Explicit consideration of salt allows for smaller box sizes to yield ensembles indistinguishable from those simulated in the largest box (Figure 8). The rule-of-thumb for a 1 nm minimum distance from the solute to the box wall holds well in this case.

#### Peptide Orientations

Overstabilization of the extended peptide conformations in the smallest boxes can be explained by the strong electrostatic interactions of the protein with its periodic image. The oppositely charged termini attract each other, and given insufficient solvent buffer between the periodic copies of the molecule, the artificial interaction with a periodic image will stabilize an extended peptide state.

**Figure 9:**
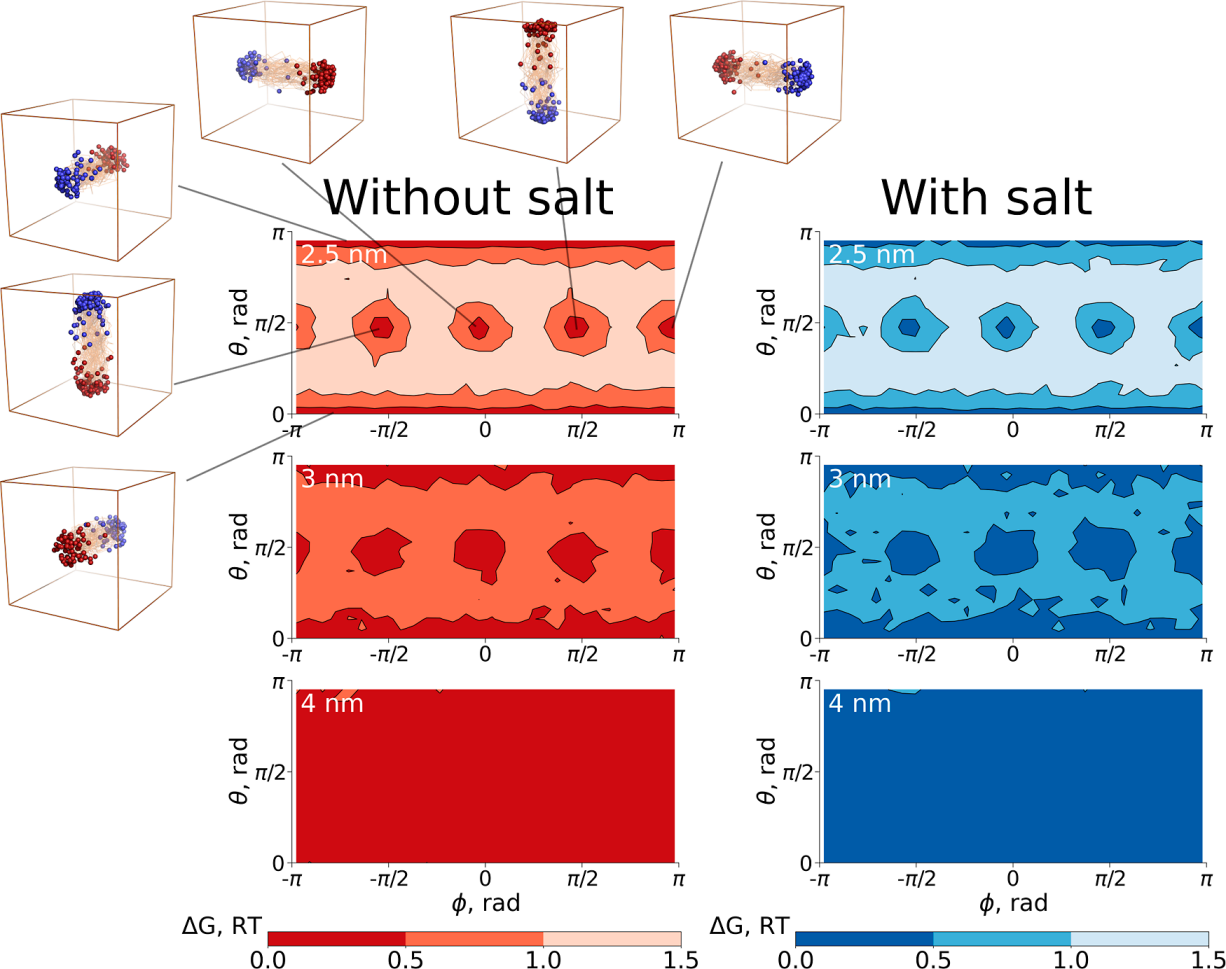
Peptide orientations mapped onto spherical coordinates for simulations in the boxes with edge lengths of 2.5, 3, and 4 nm. The orientation of a peptide was represented by a vector from N- to C-terminus. The structural ensembles illustrate orientations in the minima for the smallest box size. In these structures, the N-terminus is depicted by blue spheres and the C-terminus by red spheres.

We visualize this effect in Figure 9 for the three smallest boxes (and in Figure S3 for all box sizes), where the peptide orientations (a vector from the N- to C-terminus) were mapped onto spherical coordinates. It is evident that in the smallest boxes with an edge length of 2.5 and 3 nm, six minima in the orientational landscape emerge. These minima match with peptide N-to-C vector directions pointing to the faces of the cubic unit cell, as shown by the representative subensembles in Figure 9.

**Figure 10:**
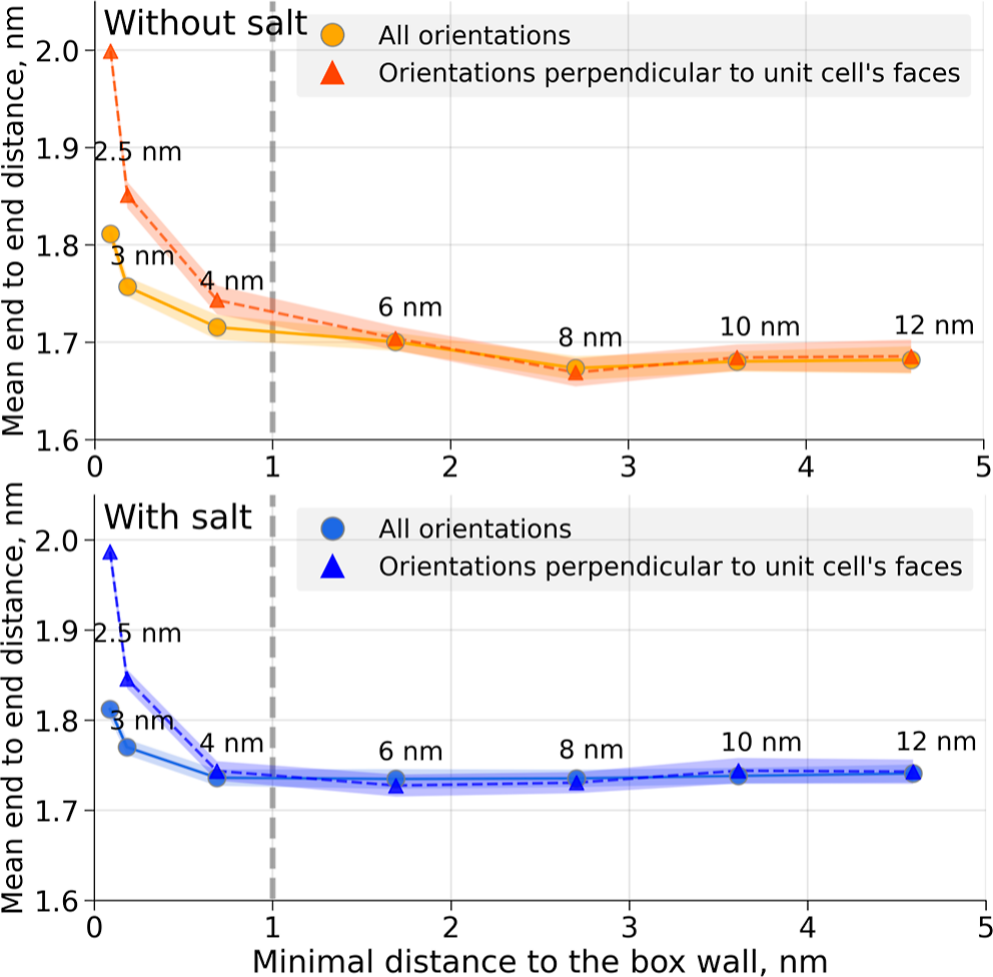
Mean end-to-end distance comparison. Averages over the whole ensemble are compared to the averages over the conformers with particular peptide orientations perpendicular to the cubic unit cell walls (minima observed in Figure 9). Simulations without salt (top panel) and with salt (bottom panel). Shaded areas correspond to the 95% confidence intervals estimated from independent simulations.

The peptide orientations that are perpendicular to the cubic unit cell’s face are stabilized in their extended conformations for the smallest boxes. This is clearly quantified by mean end-to-end distance calculations (Figure 10). The end-to-end distances averaged only over the conformers from the minima regions observed in Figure 9 are significantly longer than the averages over all conformers for the 2.5 and 3 nm boxes.

### Next-Generation Corrections

In this work, we observed that the application of correction terms for electrostatic free-energy differences might be beneficial for alchemical simulations with charged simulation boxes, while the artifacts become negligible for overall neutral simulation boxes.

In practical settings, to compute the (relative) binding free energy of charged species from molecular simulations, some considerations still need to be made before calculated binding free energies become representative of experimental values. Effectively, one can elegantly avoid the use of a charge-changing counterion by placing the actual ligand in the simulation box, as in the double-system single-box approach.^10^ For overall neutral simulation boxes, the artifacts due to the summation over discrete water molecules do not appear; however, the transfer over the vacuum–water interface (the Galvani potential) may play a role if the ligand and the protein host are very different in size, in relation to the simulation box.^7,12,21^ Furthermore, the inaccuracy of the water model to describe the dielectric constant of the salt solution could warrant the need for further corrections.

For a single ion in solution, we have previously described a restraining force to correct some of the electrostatic artifacts during a simulation.^40^ Another direction for correction development that we foresee is an efficient methodology to reweigh conformational ensembles based on corrected electrostatic interaction energies. Such an approach could, in principle, allow correcting conformational ensemble populations, ensuring that the computed free-energy profiles do not suffer from finite-size effects. This is also relevant in alchemical free-energy calculations, as calculated free energies directly depend on the sampling of the underlying ensemble. While properly correcting sampling artifacts using reweighing remains a complex challenge, our recommendation is to follow the system setup guidelines to avoid or minimize finite-size electrostatic artifacts.

An important technical challenge of the efficiency of correction calculations also needs to be addressed. As observed in the current work, solving the Poisson equation with periodic boundary conditions for large boxes and multiple charged particles (when simulations with salt were considered) for a considerable amount of configurations took a substantial amount of time (e.g., 1 h on a single CPU for the cubic box with an edge length of 8 nm). Speeding up the correction calculations would allow for their wider application in practice.

## Conclusions

We have shown that for alchemical free-energy calculations, finite-size electrostatic artifacts may occur in small charged simulation boxes when simulating without explicit salt. Corrections can be applied to reduce these artifacts. However, this comes at the cost of increased uncertainties, and not all artifacts can be alleviated. For both alchemical and conventional molecular dynamics simulations, we find converged free energies independent of the applied box size in neutral simulation boxes with a solvent buffer larger than 1 nm. In addition, we found that the inclusion of salt helps to further reduce finite-size periodicity artifacts. This leads to the following recommendations of best practice to minimize the occurrence of electrostatic finite-size artifacts: (a) setup of free-energy calculations such that there is no overall charge change, (b) ensuring an overall neutral simulation box, (c) the use of a solvent buffer of more than 1 nm, and (d) the use of salt if possible. In cases where this is not practical, for specific applications in small simulation boxes, free-energy corrections may be applied to reduce the ensuing artifacts.

## Supporting Information Available

Figures with the correction values for buckyball simulations, peptide orientations mapped onto spherical coordinates for all the investigated box sizes, table comparing free-energy estimators for the buckyball calculations, free-energy differences for the buckyball calculations, and corrections for the buckyball free-energy calculations (PDF)
