# Supplementary material for: Guidelines for Free-Energy Calculations Involving Charge Changes
Source: J Chem Theory Comput. 2024 Jan 2;20(2):914–25. doi: 10.1021/acs.jctc.3c00757 (PMC10809403; doi:10.1021/acs.jctc.3c00757)
Supplement: Supplementary file 1 — ct3c00757_si_001.pdf [file ct3c00757_si_001.pdf]

Supporting Information: Guidelines for free energy calculations involving charge changes

Drazen Petrov,† Jan Walther Perthold,† Chris Oostenbrink,†,‡ Bert L. de Groot,¶ and Vytautas Gapsys*,¶,§

†*Institute for Molecular Modeling and Simulation, Department of Material Sciences and Process Engineering, University of Natural Resources and Life Sciences, Vienna, 1190 Vienna, Austria*

‡*Christian Doppler Laboratory for Molecular Informatics in the Biosciences, University of Natural Resources and Life Sciences, Vienna, 1190 Vienna*

¶*Computational Biomolecular Dynamics Group, Department of Theoretical and Computational Biophysics, Max Planck Institute for Multidisciplinary Sciences, 37077 Göttingen, Germany*

§*Computational Chemistry, Janssen Research & Development, Janssen Pharmaceutica N. V., Turnhoutseweg 30, B-2340 Beerse, Belgium.*

E-mail: vgapsys@gwdg.de

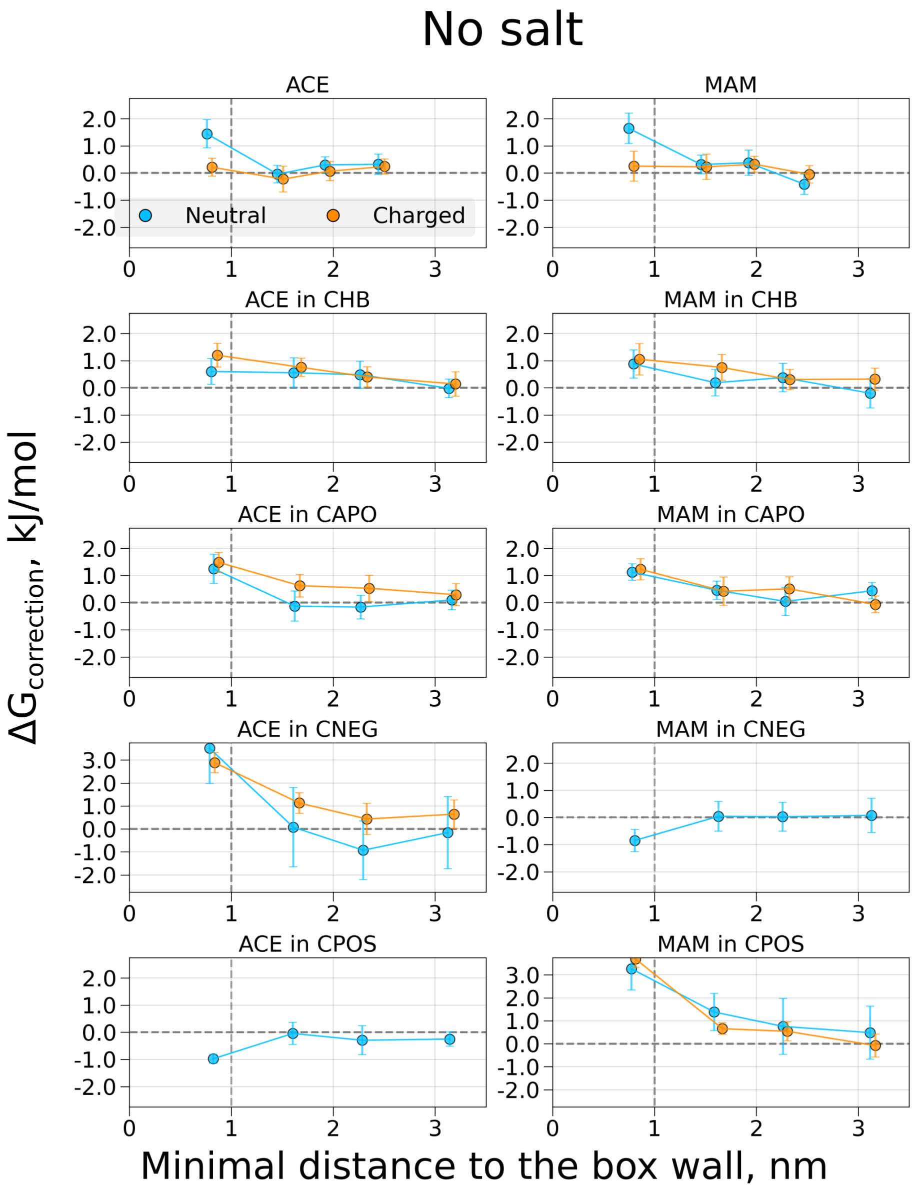

Figure S1: Corrections to the calculated ΔG for coupling electrostatic interactions of the ACE and MAM ligands and decoupling an ion in solution. Neutral (light blue) and charged (orange) systems are depicted. Simulations were performed without salt. The symbols for the charged systems are offset by 0.05 nm along x-axis for visualization purpose.

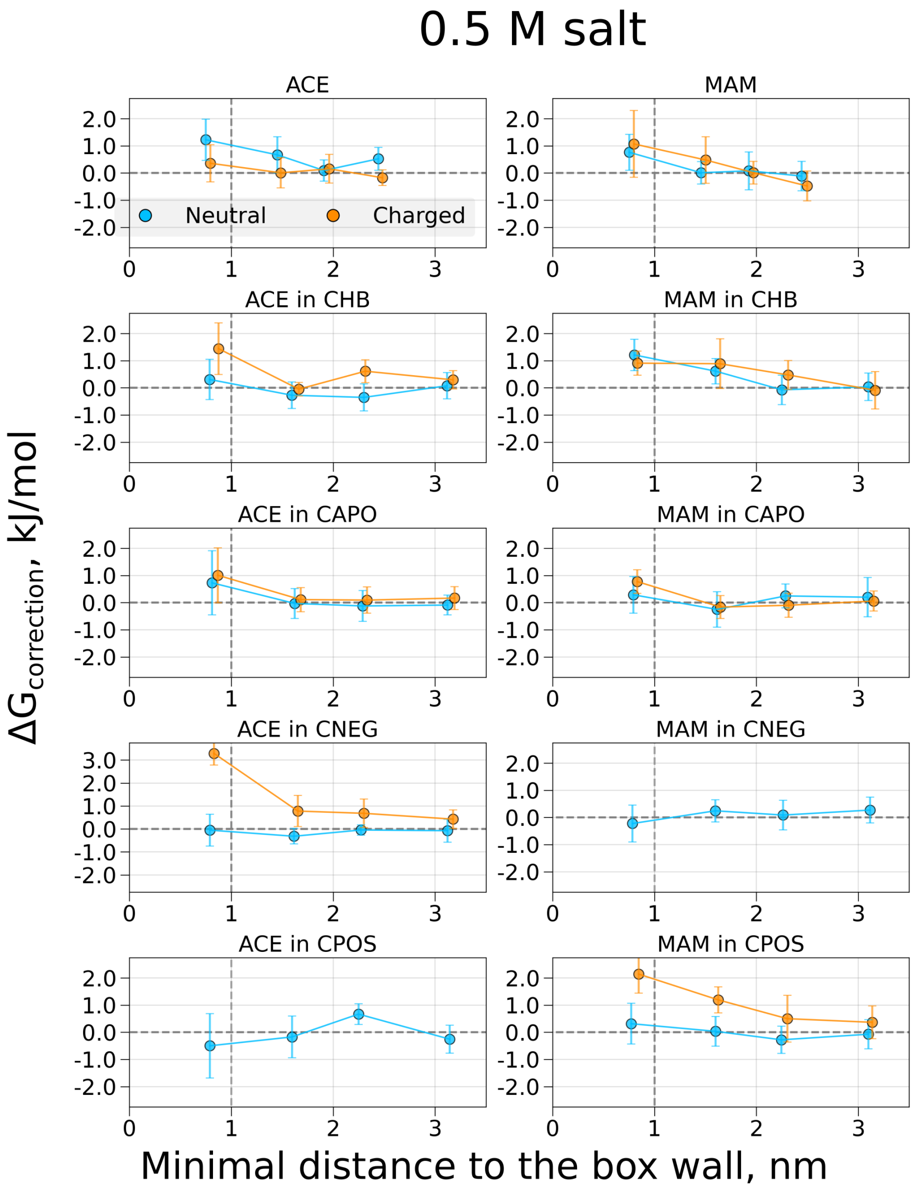

Figure S2: Corrections to the calculated ΔG for coupling electrostatic interactions of the ACE and MAM ligands and decoupling an ion in solution. Neutral (light blue) and charged (orange) systems are depicted. Simulations were performed with 0.5 M NaCl salt. The symbols for the charged systems are offset by 0.05 nm along x-axis for visualization purpose.

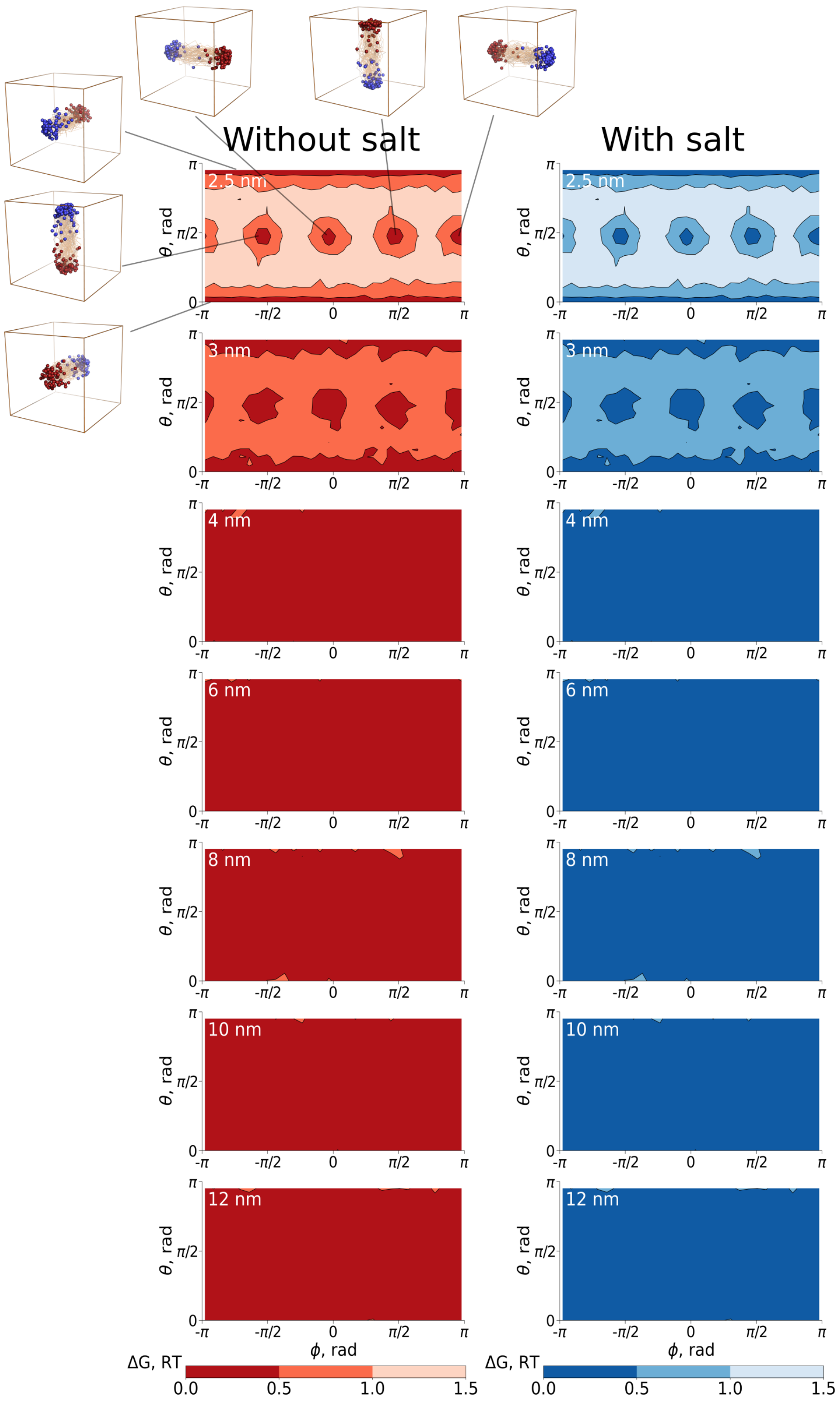

Figure S3: Peptide orientations mapped onto spherical coordinates for simulations in the boxes with edge length of 2.5, 3, 4, 6, 8, 10 and 12 nm. The orientation of a peptide was represented by a vector from N- to C-terminus. The structural ensembles illustrate orientations in the minima for the smallest box size. In these structures, the N-terminus is depicted by blue spheres, C-terminus by red spheres.

Table S1: The dependence of the calculated free energy differences on the choice of an estimator was evaluated by comparing estimates thermodynamic integration (TI) and MBAR. For TI, $\partial H/\partial\lambda$ curves were integrated using cubic splines. Three buckyball systems were considered in this comparison: ACE in CNEG, MAM in CPOS and ACE in CAPO. For this comparison we used simulations with 0.5 M salt in a charged simulation box. The values in the table are in kJ/mol. Uncertainty is calculated as a standard error from 5 independent simulations.

	ace_in_cneg		mam_in_cpos		ace_in_capo	
	TI	MBAR	TI	MBAR	TI	MBAR
box1	791.02 ± 0.4	791.09 ± 0.44	453.58 ± 0.05	453.51 ± 0.05	672.14 ± 0.44	672.44 ± 0.34
box2	791.63 ± 0.24	791.51 ± 0.14	455.12 ± 0.06	455.05 ± 0.07	673.19 ± 0.32	673.39 ± 0.37
box3	792.04 ± 0.58	792.06 ± 0.39	455.45 ± 0.04	455.38 ± 0.04	673.5 ± 0.57	673.57 ± 0.53
box4	791.97 ± 0.32	792.03 ± 0.37	455.78 ± 0.05	455.7 ± 0.05	673.74 ± 0.26	674.04 ± 0.21

Table S2: Free energy differences (kJ/mol) for buckyball systems simulated without salt retaining neutral simulation box. Box sizes defined according to the description in Figure 1 in the main text: box1 - the smallest box, box4 - largest box. Uncertainty is calculated as a standard error from 5 independent simulations.

	box1	box2	box3	box4
ace	-775.45 ± 0.47	-775.64 ± 0.43	-775.76 ± 0.24	-775.77 ± 0.08
ace_in_capo	-547.99 ± 0.1	-547.84 ± 0.05	-547.75 ± 0.06	-547.66 ± 0.05
ace_in_chb	-591.52 ± 0.1	-591.6 ± 0.09	-591.59 ± 0.08	-591.69 ± 0.08
ace_in_cneg	-1274.59 ± 0.17	-1274.08 ± 0.06	-1274.13 ± 0.08	-1274.07 ± 0.04
ace_in_cpos	443.7 ± 0.35	444.05 ± 0.16	443.76 ± 0.2	443.71 ± 0.07
mam	-1064.34 ± 0.33	-1065.6 ± 0.21	-1065.13 ± 0.35	-1065.1 ± 0.28
mam_in_capo	-889.86 ± 0.27	-889.96 ± 0.31	-889.74 ± 0.24	-890.1 ± 0.08
mam_in_chb	-924.3 ± 0.54	-924.67 ± 0.27	-925.03 ± 0.32	-924.64 ± 0.23
mam_in_cneg	86.05 ± 0.04	85.92 ± 0.08	85.86 ± 0.08	85.94 ± 0.08
mam_in_cpos	-2109.48 ± 0.12	-2109.06 ± 0.12	-2108.93 ± 0.22	-2109.0 ± 0.18

Table S3: Free energy differences with corrections (kJ/mol) for buckyball systems simulated without salt retaining neutral simulation box. Box sizes defined according to the description in Figure 1 in the main text: box1 - the smallest box, box4 - largest box. Uncertainty is calculated as a standard error from 5 independent simulations.

	box1	box2	box3	box4
ace	-774.0 ± 0.54	-775.68 ± 0.46	-775.46 ± 0.28	-775.45 ± 0.21
ace_in_capo	-546.74 ± 0.29	-547.97 ± 0.29	-547.91 ± 0.23	-547.56 ± 0.19
ace_in_chb	-590.92 ± 0.26	-591.05 ± 0.29	-591.1 ± 0.27	-591.71 ± 0.19
ace_in_cneg	-1271.08 ± 0.8	-1274.01 ± 0.88	-1275.06 ± 0.65	-1274.23 ± 0.8
ace_in_cpos	442.72 ± 0.36	444.01 ± 0.26	443.47 ± 0.34	443.46 ± 0.15
mam	-1062.7 ± 0.44	-1065.28 ± 0.28	-1064.75 ± 0.42	-1065.51 ± 0.34
mam_in_capo	-888.73 ± 0.31	-889.51 ± 0.36	-889.7 ± 0.36	-889.66 ± 0.18
mam_in_chb	-923.42 ± 0.6	-924.48 ± 0.37	-924.65 ± 0.42	-924.84 ± 0.36
mam_in_cneg	85.2 ± 0.21	85.96 ± 0.29	85.89 ± 0.28	86.02 ± 0.33
mam_in_cpos	-2106.21 ± 0.49	-2107.68 ± 0.43	-2108.17 ± 0.66	-2108.52 ± 0.62

Table S4: Free energy differences (kJ/mol) for buckyball systems simulated without salt in a charged simulation box. Box sizes defined according to the description in Figure 1 in the main text: box1 - the smallest box, box4 - largest box. Uncertainty is calculated as a standard error from 5 independent simulations.

	box1	box2	box3	box4
ace	439.44 ± 0.25	439.34 ± 0.12	439.59 ± 0.12	439.58 ± 0.09
ace_in_capo	665.61 ± 0.48	666.77 ± 0.21	667.08 ± 0.19	667.29 ± 0.11
ace_in_chb	622.47 ± 0.2	623.04 ± 0.21	623.71 ± 0.1	623.38 ± 0.12
ace_in_cneg	784.38 ± 0.32	785.59 ± 0.09	785.89 ± 0.07	785.94 ± 0.14
ace_in_cpos				
mam	150.02 ± 0.06	150.04 ± 0.04	150.24 ± 0.05	150.34 ± 0.06
mam_in_capo	324.01 ± 0.05	324.64 ± 0.06	324.97 ± 0.05	325.05 ± 0.07
mam_in_chb	288.84 ± 0.03	289.51 ± 0.07	289.91 ± 0.06	289.98 ± 0.06
mam_in_cneg				
mam_in_cpos	452.97 ± 0.08	454.52 ± 0.05	455.07 ± 0.06	455.37 ± 0.05

Table S5: Free energy differences with corrections (kJ/mol) for buckyball systems simulated without salt in a charged simulation box. Box sizes defined according to the description in Figure 1 in the main text: box1 - the smallest box, box4 - largest box. Uncertainty is calculated as a standard error from 5 independent simulations.

	box1	box2	box3	box4
ace	439.65 ± 0.3	439.12 ± 0.27	439.66 ± 0.22	439.83 ± 0.17
ace_in_capo	667.09 ± 0.51	667.39 ± 0.3	667.62 ± 0.31	667.58 ± 0.23
ace_in_chb	623.67 ± 0.3	623.8 ± 0.27	624.11 ± 0.22	623.52 ± 0.26
ace_in_cneg	787.27 ± 0.39	786.72 ± 0.24	786.33 ± 0.35	786.58 ± 0.35
ace_in_cpos				
mam	150.27 ± 0.29	150.27 ± 0.24	150.56 ± 0.16	150.29 ± 0.17
mam_in_capo	325.24 ± 0.21	325.07 ± 0.27	325.48 ± 0.23	324.98 ± 0.17
mam_in_chb	289.9 ± 0.3	290.26 ± 0.25	290.22 ± 0.2	290.3 ± 0.21
mam_in_cneg				
mam_in_cpos	456.66 ± 0.2	455.18 ± 0.14	455.61 ± 0.22	455.3 ± 0.26

Table S6: Free energy differences (kJ/mol) for buckyball systems simulated with 0.5 M salt retaining neutral simulation box. Box sizes defined according to the description in Figure 1 in the main text: box1 - the smallest box, box4 - largest box. Uncertainty is calculated as a standard error from 5 independent simulations.

	box1	box2	box3	box4
ace	-778.15 ± 0.61	-777.6 ± 0.24	-778.09 ± 0.15	-778.14 ± 0.07
ace_in_capo	-549.68 ± 0.15	-549.64 ± 0.07	-549.69 ± 0.1	-549.67 ± 0.09
ace_in_chb	-593.57 ± 0.21	-593.68 ± 0.08	-593.53 ± 0.11	-593.41 ± 0.17
ace_in_cneg	-431.33 ± 0.12	-431.6 ± 0.14	-431.47 ± 0.09	-431.45 ± 0.1
ace_in_cpos	450.88 ± 0.55	450.88 ± 0.6	451.62 ± 0.22	451.56 ± 0.34
mam	-1073.57 ± 0.52	-1073.27 ± 0.41	-1074.12 ± 0.48	-1073.51 ± 0.29
mam_in_capo	-897.29 ± 0.4	-897.54 ± 0.46	-897.19 ± 0.5	-897.66 ± 0.33
mam_in_chb	-932.25 ± 0.42	-932.54 ± 0.13	-932.15 ± 0.48	-932.16 ± 0.35
mam_in_cneg	87.98 ± 0.11	87.66 ± 0.09	87.82 ± 0.09	87.47 ± 0.04
mam_in_cpos	-767.59 ± 0.76	-767.85 ± 0.3	-768.1 ± 0.21	-767.68 ± 0.6

Table S7: Free energy differences with corrections (kJ/mol) for buckyball systems simulated with 0.5 M salt retaining neutral simulation box. Box sizes defined according to the description in Figure 1 in the main text: box1 - the smallest box, box4 - largest box. Uncertainty is calculated as a standard error from 5 independent simulations.

	box1	box2	box3	box4
ace	-776.92 ± 0.72	-776.93 ± 0.42	-778.0 ± 0.25	-777.61 ± 0.23
ace_in_capo	-548.95 ± 0.62	-549.67 ± 0.29	-549.81 ± 0.31	-549.75 ± 0.21
ace_in_chb	-593.26 ± 0.43	-593.95 ± 0.26	-593.89 ± 0.28	-593.33 ± 0.3
ace_in_cneg	-431.39 ± 0.38	-431.92 ± 0.22	-431.52 ± 0.15	-431.53 ± 0.27
ace_in_cpos	450.39 ± 0.82	450.71 ± 0.72	452.29 ± 0.29	451.31 ± 0.43
mam	-1072.8 ± 0.62	-1073.26 ± 0.46	-1074.04 ± 0.6	-1073.63 ± 0.4
mam_in_capo	-897.0 ± 0.52	-897.79 ± 0.56	-896.94 ± 0.55	-897.45 ± 0.5
mam_in_chb	-931.04 ± 0.51	-931.93 ± 0.27	-932.22 ± 0.56	-932.13 ± 0.43
mam_in_cneg	87.76 ± 0.36	87.91 ± 0.23	87.91 ± 0.29	87.74 ± 0.25
mam_in_cpos	-767.28 ± 0.85	-767.81 ± 0.41	-768.38 ± 0.33	-767.75 ± 0.66

Table S8: Free energy differences (kJ/mol) for buckyball systems simulated with 0.5 M salt in a charged simulation box. Box sizes defined according to the description in Figure 1 in the main text: box1 - the smallest box, box4 - largest box. Uncertainty is calculated as a standard error from 5 independent simulations.

	box1	box2	box3	box4
ace	446.0 ± 0.38	445.77 ± 0.26	444.95 ± 0.14	445.69 ± 0.25
ace_in_capo	672.44 ± 0.34	673.39 ± 0.37	673.57 ± 0.53	674.04 ± 0.21
ace_in_chb	629.73 ± 0.4	630.22 ± 0.36	629.57 ± 0.65	630.13 ± 0.28
ace_in_cneg	791.09 ± 0.44	791.51 ± 0.14	792.06 ± 0.39	792.03 ± 0.37
ace_in_cpos				
mam	150.23 ± 0.08	150.42 ± 0.13	150.48 ± 0.1	150.45 ± 0.1
mam_in_capo	325.1 ± 0.13	326.0 ± 0.07	326.11 ± 0.1	326.42 ± 0.14
mam_in_chb	290.09 ± 0.1	290.8 ± 0.08	291.0 ± 0.08	291.12 ± 0.05
mam_in_cneg				
mam_in_cpos	453.51 ± 0.05	455.05 ± 0.07	455.38 ± 0.04	455.7 ± 0.05

Table S9: Free energy differences with corrections (kJ/mol) for buckyball systems simulated with 0.5 M salt in a charged simulation box. Box sizes defined according to the description in Figure 1 in the main text: box1 - the smallest box, box4 - largest box. Uncertainty is calculated as a standard error from 5 independent simulations.

	box1	box2	box3	box4
ace	446.36 ± 0.51	445.77 ± 0.39	445.1 ± 0.31	445.52 ± 0.29
ace_in_capo	673.45 ± 0.62	673.5 ± 0.43	673.66 ± 0.59	674.21 ± 0.3
ace_in_chb	631.18 ± 0.63	630.17 ± 0.38	630.18 ± 0.68	630.42 ± 0.33
ace_in_cneg	794.38 ± 0.51	792.29 ± 0.37	792.74 ± 0.5	792.46 ± 0.42
ace_in_cpos				
mam	151.31 ± 0.63	150.9 ± 0.46	150.49 ± 0.24	149.98 ± 0.3
mam_in_capo	325.87 ± 0.26	325.84 ± 0.23	326.02 ± 0.25	326.48 ± 0.23
mam_in_chb	291.0 ± 0.25	291.69 ± 0.47	291.48 ± 0.28	291.03 ± 0.35
mam_in_cneg				
mam_in_cpos	455.66 ± 0.36	456.24 ± 0.26	455.88 ± 0.44	456.07 ± 0.31

Table S10: Corrections (kJ/mol) for buckyball systems simulated without salt in a neutral simulation box. Box sizes defined according to the description in Figure 1 in the main text: box1 - the smallest box, box4 - largest box. Uncertainty is calculated as a standard error from 5 independent simulations.

	box1	box2	box3	box4
ace	1.45 ± 0.27	-0.04 ± 0.16	0.3 ± 0.15	0.32 ± 0.19
ace_in_capo	1.25 ± 0.27	-0.13 ± 0.28	-0.16 ± 0.22	0.1 ± 0.18
ace_in_chb	0.6 ± 0.24	0.56 ± 0.28	0.48 ± 0.26	-0.02 ± 0.17
ace_in_cneg	3.52 ± 0.78	0.08 ± 0.88	-0.93 ± 0.65	-0.16 ± 0.8
ace_in_cpos	-0.98 ± 0.08	-0.04 ± 0.21	-0.29 ± 0.27	-0.25 ± 0.13
mam	1.65 ± 0.29	0.31 ± 0.18	0.38 ± 0.24	-0.41 ± 0.19
mam_in_capo	1.13 ± 0.16	0.46 ± 0.17	0.05 ± 0.27	0.44 ± 0.16
mam_in_chb	0.88 ± 0.27	0.19 ± 0.25	0.38 ± 0.27	-0.2 ± 0.28
mam_in_cneg	-0.85 ± 0.21	0.04 ± 0.28	0.03 ± 0.27	0.08 ± 0.32
mam_in_cpos	3.27 ± 0.47	1.39 ± 0.41	0.76 ± 0.62	0.49 ± 0.59

Table S11: Corrections (kJ/mol) for buckyball systems simulated without salt in a charged simulation box. Box sizes defined according to the description in Figure 1 in the main text: box1 - the smallest box, box4 - largest box. Uncertainty is calculated as a standard error from 5 independent simulations.

	box1	box2	box3	box4
ace	0.21 ± 0.17	-0.22 ± 0.24	0.07 ± 0.18	0.24 ± 0.14
ace_in_capo	1.49 ± 0.19	0.63 ± 0.21	0.53 ± 0.24	0.29 ± 0.2
ace_in_chb	1.2 ± 0.22	0.76 ± 0.17	0.4 ± 0.19	0.14 ± 0.23
ace_in_cneg	2.89 ± 0.22	1.13 ± 0.23	0.43 ± 0.35	0.64 ± 0.32
ace_in_cpos				
mam	0.25 ± 0.28	0.23 ± 0.24	0.33 ± 0.15	-0.05 ± 0.16
mam_in_capo	1.23 ± 0.2	0.42 ± 0.27	0.51 ± 0.23	-0.06 ± 0.16
mam_in_chb	1.06 ± 0.3	0.75 ± 0.24	0.31 ± 0.19	0.32 ± 0.21
mam_in_cneg				
mam_in_cpos	3.69 ± 0.19	0.66 ± 0.13	0.54 ± 0.21	-0.08 ± 0.25

Table S12: Corrections (kJ/mol) for buckyball systems simulated with 0.5 M salt in a neutral simulation box. Box sizes defined according to the description in Figure 1 in the main text: box1 - the smallest box, box4 - largest box. Uncertainty is calculated as a standard error from 5 independent simulations.

	box1	box2	box3	box4
ace	1.23 ± 0.39	0.67 ± 0.34	0.09 ± 0.2	0.53 ± 0.22
ace_in_capo	0.73 ± 0.6	-0.03 ± 0.28	-0.12 ± 0.29	-0.09 ± 0.19
ace_in_chb	0.31 ± 0.38	-0.27 ± 0.25	-0.35 ± 0.25	0.08 ± 0.25
ace_in_cneg	-0.05 ± 0.36	-0.32 ± 0.17	-0.04 ± 0.12	-0.08 ± 0.25
ace_in_cpos	-0.5 ± 0.61	-0.17 ± 0.39	0.67 ± 0.19	-0.25 ± 0.26
mam	0.77 ± 0.34	0.01 ± 0.21	0.08 ± 0.36	-0.11 ± 0.28
mam_in_capo	0.29 ± 0.34	-0.25 ± 0.33	0.25 ± 0.22	0.2 ± 0.37
mam_in_chb	1.21 ± 0.29	0.61 ± 0.24	-0.07 ± 0.28	0.04 ± 0.26
mam_in_cneg	-0.22 ± 0.35	0.25 ± 0.21	0.09 ± 0.28	0.27 ± 0.24
mam_in_cpos	0.32 ± 0.38	0.04 ± 0.28	-0.28 ± 0.26	-0.07 ± 0.27

Table S13: Corrections (kJ/mol) for buckyball systems simulated with 0.5 M salt in a charged simulation box. Box sizes defined according to the description in Figure 1 in the main text: box1 - the smallest box, box4 - largest box. Uncertainty is calculated as a standard error from 5 independent simulations.

	box1	box2	box3	box4
ace	0.36 ± 0.35	0.01 ± 0.28	0.16 ± 0.27	-0.17 ± 0.15
ace_in_capo	1.01 ± 0.52	0.11 ± 0.23	0.09 ± 0.25	0.17 ± 0.21
ace_in_chb	1.45 ± 0.49	-0.05 ± 0.13	0.61 ± 0.21	0.3 ± 0.17
ace_in_cneg	3.29 ± 0.26	0.78 ± 0.35	0.68 ± 0.32	0.43 ± 0.21
ace_in_cpos				
mam	1.07 ± 0.63	0.48 ± 0.44	0.01 ± 0.21	-0.48 ± 0.28
mam_in_capo	0.78 ± 0.22	-0.16 ± 0.22	-0.1 ± 0.23	0.06 ± 0.19
mam_in_chb	0.91 ± 0.23	0.89 ± 0.47	0.48 ± 0.27	-0.09 ± 0.35
mam_in_cneg				
mam_in_cpos	2.15 ± 0.36	1.2 ± 0.25	0.5 ± 0.44	0.37 ± 0.31
